# The impact of healthcare system quality and economic factors on the older adult population: a health economics perspective

**DOI:** 10.3389/fpubh.2024.1454699

**Published:** 2024-10-17

**Authors:** Iulia Cristina Iuga, Raluca Andreea Nerişanu, Horia Iuga

**Affiliations:** ^1^Department of Finance and Accounting, "1 Decembrie 1918" University of Alba Iulia, Alba Iulia, Romania; ^2^Department of Finance and Accounting, University Lucian Blaga of Sibiu, Sibiu, Romania; ^3^Faculty of Medicine (Student), "Iuliu Hațieganu" University of Medicine and Pharmacy, Cluj- Napoca, Romania

**Keywords:** healthcare system capabilities, macroeconomic effects, economics of the older adult, wavelet coherence analysis, system GMM, European Union demographics

## Abstract

**Purpose:**

This study investigates the influence of economic conditions, healthcare system capacity, and health-related variables on the proportion of the older adult population (Population ages 65 and above) in European Union countries. It aims to identify how factors such as GDP, unemployment, inflation, healthcare expenditure, hospital bed availability, and the prevalence of chronic diseases impact the aging demographic.

**Methods:**

This study explores the dynamic interactions and temporal relationships between economic stability, healthcare capacity, chronic disease prevalence, and demographic aging patterns. The research employs a mixed-method approach, utilizing System GMM and wavelet coherence analysis on panel data from 27 EU countries between 2000 and 2021.

**Results:**

The findings reveal significant positive associations between economic prosperity and healthcare resources with the size of the older adult population. Increased GDP, efficient healthcare spending, and hospital bed availability are positively correlated with a larger older adult demographic. In contrast, high unemployment and inflation are linked to negative outcomes for the older adult population, reducing available resources and access to healthcare. Wavelet coherence analysis further uncovers how fluctuations in the prevalence of chronic diseases influence aging trends across different periods and frequencies.

**Conclusion:**

The study highlights the importance of integrated economic and healthcare policies to support the growing older adult population. Ensuring economic stability, enhancing healthcare infrastructure, and effectively managing chronic diseases are essential for improving quality of life and promoting sustainable aging in EU societies.

## 1 Introduction

The aging population in the European Union (EU) presents significant challenges for public health, economic stability, and social welfare. As the number of individuals aged 65 and above increases, it is crucial to develop effective strategies that address the healthcare and economic needs of this demographic to ensure sustainable social and healthcare systems.

Economic factors such as Gross Domestic Product (GDP) *per capita*, unemployment rate, and inflation are fundamental determinants of a country’s ability to support its aging population (1).

GDP, measuring a country’s total economic output, significantly impacts citizens’ economic health and living standards, especially for the older adult. Countries with strong GDPs can more effectively fund healthcare, social services, and pensions, benefiting their senior population (2). High GDP countries often boast well-funded health systems capable of providing extensive care and assistance to the older adult, thereby enhancing both their longevity and quality of life, and increasing the older adult proportion in society (3).

Job market conditions significantly impact the older adult. Elevated unemployment levels pressure state budgets, leading to reduced essential services and healthcare for seniors. In contrast, low unemployment stabilizes funding for healthcare and pensions, supporting a conducive environment for older adult well-being (4). Additionally, inflation affects seniors, especially those relying on fixed incomes, by making critical services more costly. Yet, when inflation is kept in check, it enhances healthcare accessibility for the older adult, positively affecting their health and increasing their share in the population (5).

Regarding healthcare system capabilities, the number of available hospital beds is essential. Adequate hospital bed capacity is essential for the older adult’s healthcare, who need more medical attention. Insufficient beds lead to extended wait times and reduced care quality, harming seniors’ health. Additionally, the GDP share for healthcare significantly affects outcomes, with higher spending crucial for prevention, treatment, and maintaining older adult well-being. Countries dedicating a larger GDP share to healthcare are typically more adept at addressing the needs of their aging population, leading to a higher older adult population ratio (6).

In many countries, chronic diseases account for a significant portion of healthcare expenditures, which limits the government’s ability to invest in other areas of public health or in general infrastructure (7).

The high prevalence of chronic diseases among the older adult significantly escalates healthcare costs. The selection of Diabetes Mellitus, circulatory diseases, and respiratory diseases for this study is based on their high prevalence and significant impact on the older adult population across Europe. Together, they represent the most common and severe health challenges faced by older adults in Europe, accounting for a large proportion of healthcare utilization and mortality in this demographic. According to Eurostat (8), circulatory diseases accounted for 32.4% of all deaths in the EU in 2021, making them the leading cause of mortality. Deaths due to Diabetes Mellitus reached 25.56% (9), while respiratory diseases contributed to around 6.7% of hospitalizations among those aged 65 and over (10). These statistics underscore the urgent need for targeted healthcare strategies.

The interplay between economic factors, healthcare system capabilities, and health-related variables has a profound impact on the older adult population, particularly those with chronic diseases.

This study strategically focuses on three key groups of indicators (economic factors, healthcare system capabilities, and health-related variables) due to their significant influence on the older adult population. Economic factors impact the financial resources available for older adult care (10), healthcare system capabilities evaluate the infrastructure and quality of services (10), and health-related variables highlight specific health challenges faced by the older adult (11). By integrating these indicators, the study provides a comprehensive view of the factors shaping the well-being and demographic trends of the older adult in the EU, enabling the development of targeted policies and interventions to address aging-related challenges effectively.

This article aims to analyze the influence of three groups of indicators (economic factors, healthcare system capabilities, and health-related variables) on the proportion of the population aged 65 and over in EU member countries. By examining the interaction between economic indicators (GDP, unemployment rate, and inflation), healthcare system capabilities (hospital bed availability, health spending as a percentage of GDP), and health-related variables (prevalence of Diabetes Mellitus, circulatory diseases, and respiratory diseases), the study provides key insights into how these factors collectively affect the demographic aging process. Based on the context provided we state the research question: “*How do healthcare system capabilities, economic factors, and health-related variables impact the proportion of the population aged 65 and over in EU member countries?*” This study explores the intricate dynamics between these factors, shedding light on the underlying drivers of population aging and informing the development of effective strategies to tackle the associated challenges.

Utilizing advanced techniques like System GMM and wavelet coherence analysis, this study reveals the complex temporal and frequency-dependent interactions among economic stability, healthcare capacity, and chronic disease prevalence, shaping older adult well-being and demographics. The findings emphasize the need for integrated policies to address interconnected economic, healthcare, and health challenges in managing an aging population.

The novelty of this study lies in its integrative approach, combining economic factors, healthcare infrastructure, and medical variables to assess their collective impact on the older adult population. Unlike previous research that has often considered these elements separately, this study examines the economic context, healthcare system capacity, and chronic diseases in tandem, providing a comprehensive perspective on their interactions. By bridging the economic, medical, and infrastructural aspects, the study offers a deeper understanding of how these interconnected factors influence the demographic trends and well-being of the older adult population.

## 2 Literature review

### 2.1 Economic factors and older adult population nexus

Kenneth Arrow’s (12) work is one of the first to apply economic theory to the analysis of the healthcare sector, highlighting the unique challenges of the health market. This theoretical framework examines how economic factors such as GDP, unemployment, and inflation influence the allocation of healthcare resources, being crucial for assessing the economic impact of population aging on health and social care systems. The economic factors influencing health policies and the management of healthcare services encompass complex interactions between GDP, inflation, and unemployment. Phelps (13) provides a detailed analysis of how economic principles are applied within health systems, highlighting how these economic variables shape decisions regarding resource allocation and the efficiency of healthcare services.

GDP crucially affects the older adult population’s proportion by influencing a nation’s ability to support its aging citizens. As an indicator of economic health, a higher GDP enables more substantial investments in public services crucial for the older adult, like healthcare, pensions, and social supports (14). These investments improve older adult life quality, health outcomes, and longevity, increasing their population share. Strong economies can maintain and start older adult-focused programs, enhancing health and independence (15). Conversely, economic slowdowns restrict resources for older adult care, leading to cuts in services and pensions, affecting their quality of life and health (16). Such conditions can heighten financial insecurity and health risks among the older adult, reducing their life expectancy and population proportion.

The Unemployment Rate significantly impacts the older adult population by affecting economic stability and the sustainability of social security and healthcare systems. High unemployment rate can lead to economic instability, diminishing public funds essential for older adult support. Specifically, high unemployment reduces contributions to pension systems, risking lower pension payouts and affecting the older adult’s ability to afford basic needs (11). Additionally, it decreases government tax revenues, potentially resulting in healthcare budget cuts. This scenario could prolong treatment wait times and limit access to essential medications for the older adult, adversely affecting their health and possibly shortening life spans (17). Furthermore, high unemployment rate increases financial pressure on families, potentially diminishing their capacity to care for older adult members and heightening demand for already strained public services. On the flip side, low unemployment rate fosters economic stability, ensuring consistent funding for pensions and healthcare, thereby improving life expectancy and increasing the older adult’s proportion in the population. Thus, unemployment rate is pivotal in shaping the welfare and quality of life for the older adult, with its effects echoing through pension and healthcare systems (18).

Inflation significantly impacts older adult individuals, eroding their financial security, healthcare access, and overall quality of life. Rising prices diminish the purchasing power of fixed incomes, like pensions or savings, on which many older adult depend. This reduction in financial resources forces them to allocate more to escalating healthcare costs, restricting their ability to afford other necessities (19). In extreme cases, this may lead to difficult decisions between healthcare and basic needs, detrimentally affecting their health. Additionally, inflation challenges pension sustainability, as increasing living costs outpace pension adjustments, degrading older adult living standards (20). Higher healthcare expenses (21) result in longer waiting periods, decreased access to care, and greater out-of-pocket costs for the older adult, further compromising their health (22). Moreover, inflation reduces family ability to provide elder care, increasing the demand for public services. Policy responses to sustained inflation, like raising interest rates, offer mixed outcomes for the older adult, possibly boosting savings returns but also potentially slowing economic growth and affecting employment, indirectly influencing elder support mechanisms (23). Addressing the challenges inflation presents to the older adult necessitates comprehensive policies focused on pension adequacy, healthcare affordability, and economic stability to uphold an aging population’s support.

### 2.2 Optimizing healthcare capacity: the role of hospital beds and GDP health spending in supporting the older adult population

Some authors (24–28) explore various aspects of demographic aging, ranging from the application of positive aging theories to neurocognitive mechanisms and the socio-economic challenges posed by an aging population. The Theory of Demographic Aging Stages (29, 30) provides a useful conceptual framework for understanding and addressing the challenges that population aging poses to society. The Late Post-Transitional Stage represents the final phase of demographic transition and is characterized by a very high proportion of older adult individuals (65+ years) and a significant decline in the young population (under 15 years). This stage brings major economic, social, and public health challenges, profoundly impacting the structure and functioning of society. In his work, Bao (24) provides an overview of demographic transition theories, highlighting how they influence population structure in the post-transitional stage.

The healthcare system’s capacity is crucial for addressing the needs of those over 65, who often require more medical care due to a higher incidence of chronic illnesses like diabetes, cardiovascular diseases, and respiratory conditions. As the older adult population grows, so does the strain on healthcare resources, including hospital beds, medical personnel, and access to specialized treatments.

Hospital bed availability profoundly affects older adult healthcare access and demographic representation, making sufficient provision key to addressing aging challenges and supporting older adult well-being in society (31).

The availability of hospital beds is crucial for older adult healthcare quality, affecting their demographic presence. Bed density per thousand people reflects a system’s capacity for timely, adequate care, vital for older adult health needs (32). Higher bed density leads to better older adult healthcare access and outcomes, enhancing longevity and quality of life, and thus their population ratio. Conversely, bed shortages result in overcrowding, delays, and reduced care quality, harming older adult health and potentially decreasing their demographic share. The allocation of hospital beds underlines a system’s priorities in meeting older adult needs, including chronic condition management and recovery care (33). Investing in hospital beds and infrastructure is essential to meet the aging population’s needs, ensuring adequate care to improve and prolong their lives (34).

Health spending crucially affects older adult health, life quality, and independence, underscoring its role in upholding healthcare systems for an aging population. It signifies a country’s commitment to essential healthcare services, from prevention to rehabilitation, tailored to senior citizens’ needs (35). Higher health spending results in improved healthcare services, better medical care access, and the adoption of cutting-edge treatments. This enhances chronic disease management among the older adult, lowers death rates from manageable conditions, and boosts overall health and longevity. Countries dedicating a larger share of GDP to healthcare tend to see an increase in the older adult demographic, thanks to longer life spans and enhanced life quality (36).

Furthermore, sufficient health funding equips systems to offer extensive older adult care, such as long-term and home care services, crucial for their independence and dignity. Conversely, low health investment leads to healthcare access issues, delayed treatments, and a lack of older adult- specific care, negatively influencing older adults’ health and possibly reducing life expectancy (37).

Health spending also lessens the economic strain of aging populations by mitigating the costs linked to chronic and age-related diseases, fostering a healthier, more engaged older adult community. Thus, healthcare investment is vital for maintaining older adult well-being and supporting demographic sustainability.

### 2.3 The role of chronic disease (such as the prevalence of Diabetes Mellitus, diseases of the circulatory and respiratory systems) in older adult care

The prevalence of chronic diseases (Diabetes Mellitus, circulatory, and respiratory diseases) significantly affects the older adult population’s size and health, influencing mortality rates, healthcare demand, and overall well-being (38). Diabetes Mellitus requires continuous care and lifestyle changes to prevent complications, with its high prevalence necessitating more healthcare services and potentially increasing older adult mortality (39). Similarly, circulatory diseases demand significant medical resources for management, affecting older adult life expectancy and demographic proportions. Preventive and management strategies are crucial for mitigating these impacts (40). Respiratory diseases also pose severe risks, impairing physical function and increasing healthcare needs, with effective prevention and management essential for reducing their prevalence and mortality impact (41). Addressing these conditions through comprehensive healthcare planning and preventive measures is key to enhancing the older adult’s health and longevity, ensuring they maintain a significant share of the population.

The prevalence of the three chronic diseases (Diabetes Mellitus, circulatory diseases, and respiratory diseases) has significant implications for both economic indicators and healthcare system capabilities. High prevalence rates of chronic diseases place a substantial financial burden on healthcare systems due to the need for continuous treatment, management, and hospitalization (42). This, in turn, leads to increased healthcare expenditures, and in some countries, resources may be diverted from other productive sectors of the economy.

Moreover, the rising demand for medical services and medications can drive up healthcare costs, contributing to inflationary pressures. As the cost of medical care, pharmaceuticals, and hospital services rises, overall consumer prices may also increase, particularly in economies where healthcare spending constitutes a substantial portion of household expenditures (43). This can reduce disposable income and negatively impact the broader economy.

Chronic diseases also have a significant impact on labor force participation, increasing absenteeism and leading to early retirement due to health complications (44). This reduces the available workforce and, consequently, labor productivity. For example, some studies (45–47) show that individuals with diabetes have higher rates of absenteeism and an increased risk of early retirement due to health-related reasons. Additionally, individuals suffering from chronic conditions may face challenges in maintaining stable employment, further straining social security systems and increasing demand for public healthcare services.

Regarding healthcare system capabilities, high prevalence of chronic diseases increases the demand for hospital admissions, often resulting in a shortage of available beds. This can lead to overcrowded hospitals, longer waiting times, and diminished quality of care (42). Inadequate hospital bed availability exacerbates the healthcare system’s inability to meet acute medical needs, especially in countries with limited healthcare infrastructure.

Furthermore, as the prevalence of chronic diseases rises, healthcare spending often increases to accommodate the necessary treatments, hospitalizations, and preventive measures. This rise in spending reflects the growing economic burden of managing chronic conditions (42).

In the existing literature, studies have been found that explore the individual influence of economic factors, healthcare system capacities, or the prevalence of chronic diseases on the well-being of the older adult population, as reflected in various dimensions of this demographic group. However, we did not find comprehensive research on their cumulative influence. This gap highlights the need for an integrated analysis to understand the complex interplay between these factors and their collective impact on the older adult demographic in European Union countries.

This article introduces groundbreaking insights through an integrated analysis of how economic factors (such as GDP, unemployment, and inflation), healthcare system capacities (notably hospital bed availability and health spending), and the prevalence of chronic diseases collectively affect the quality of life and life expectancy of the older adult. It uniquely explores the intertwined impact of these elements on the proportion of the older adult population, offering a holistic view previously unexamined in depth. It further delves into the direct influence of economic health on essential services for the older adult, highlighting the critical role of healthcare infrastructure and chronic disease management. These innovative contributions provide a comprehensive understanding of the variables shaping the well-being of the aging population.

## 3 Methods

The rationale for selecting these three groups of indicators (economic factors, healthcare system capabilities, and health-related variables) is that they provide direct economic and systemic insights essential for assessing the capacity of healthcare systems and economic conditions to support an aging population. These indicators allow for a focused evaluation of the structural and financial dimensions critical for addressing the needs of the older adult.

To analyze the influence of economic indicators and health system capacities on the proportion of older adult population, we have obtained the annual data of each of the variables explained in Table 1, on a period of 21 years, from 2000 to 2021, for a total of 27 European countries, thus constructing a panel data.

**Table 1:** Variables and data sources.

Symbol/variables	Description/unit	Data source
Dependent		
Older adult population (POP65)	Population ages 65 and above-percent of total	World Bank, https://data.worldbank.org/indicator/SP.POP.65UP.TO.ZS?view=chat
Core explanatory		
Economic factors		
GDP (GDP)	GDP *per capita*, Purchasing Power Parity	World Bank, https://data.worldbank.org/indicator/NY.GDP.PCAP.PP.CD
Unemployment rate (UR)	Unemployment, total (% of total labor force)	World Bank, https://data.worldbank.org/indicator/SL.UEM.TOTL.ZS
Inflation (INF)	Inflation: percent change in the Consumer Price Index	World Bank, https://data.worldbank.org/indicator/FP.CPI.TOTL.ZG?view=chart
Healthcare system capabilities		
Hospital beds (HOSP-B)	The number of hospital beds per 1,000 residents of the country.	OECD, https://data.oecd.org/healtheqt/hospital-beds.htm
Health spending as percent of GDP. (HS/GDP)	Level of current health expenditure expressed as a percentage of GDP.	World Bank, https://data.worldbank.org/indicator/SH.XPD.CHEX.GD.ZS
Control variables		
Health-related variables		
Diabetes Mellitus	In-patients, total number	Eurostat, https://ec.europa.eu/eurostat/databrowser/view/hlth_co_disch1custom_8820332/default/table
Circulatory diseases	In-patients, total number	
Respiratory diseases	In-patients, total number	

Firstly, in the results section, the descriptive statistics were exposed and analyzed. Descriptive statistics include Mean, Median, Maximum value, Minimum value, and Std. Dev., Skewness. After the descriptive statistics interpretation, Dickey Fuller unit root tests were performed, in order to test the stationarity of each series (48). Next heteroskedasticity test of the residuals were performed in order to identify which type of regression models can be applied on the data. The heteroskedasticity tests applied were Glejser and White tests. Pearson correlation was performed along the results variables in order to identify the causal relationships among different variables and to prepare the data for the modeling phase and wavelet coherence.

In 1982 (49), Hansen proposes the General Method of Moments (GMM) estimator, for systems that present more moment conditions than model parameters (50). We will use the GMM method to estimate the most significant estimator for the effect of the health infrastructure variables and welfare indicators on the proportion of the older adult people. The estimator of the GMM follows the next methodology.

Firstly, in Zsohar (51), the estimation of the general method of moments is presenting for an observed sample of form $\left\{\mathrm{xi}:i=1,2,\ldots ,n\right\}$ extracted from a normal population $X\sim N\left(\mu ,\sigma^{2}\right)$, where *μ* is the population mean and *σ^2^* is the variance.

$E\;\left(x^{k}\right)$ is the *k*’th theoretical moment of the population $N\;\left(\mu ,\sigma^{2}\right),k=1,2\text{,}$

$E\;\left[{\left(x-\mu \right)}^{k}\right]$ is the *k*’th theoretical moment about the mean, of the population $N\;\left(\mu ,\sigma^{2}\right),k=1,2\text{,}$

Therefore, the corresponding *k*th sample moments are given by the Equation 1:


(1) $$\begin{array}{l} g_{k}=\frac{1}{n}\overset{n}{\underset{i=1}{\sum }}x_{i}^{k},k=1,2.\ldots \end{array}$$

Also, the *k*’th corresponding sample moment about the mean is given by Equation 2:


(2) $$g_{k}^{*}=\frac{1}{n}\overset{n}{\underset{i=1}{\sum }}\left(x_{i}-\hat{\mu }\right),k=1,2\ldots$$

Where $\hat{\mu }$ is the point estimator of the population mean *μ*.

If we define the criterion function Qn ($\hat{\mu }$) as


(3) $$Q_{n}\left(\hat{\mu }\right)={g_{k}}^{\prime }\cdot W_{n}\cdot g$$

*W_n_* is the weighting matrix, that coverages the positive definitive matrix *W* as *n* grows large (Equation 3).

The GMM estimator is given by Equation 4:


(4) $$\hat{g}=\arg\min g\;|\;g\in \Theta$$

The method of moments becomes the general method of moments if the number of moments exceeds the number of parameters.

Next, wavelet coherence is calculated, plotted and interpreted. Wavelet analysis offers the benefit of breaking down a time series into simpler functions that encompass valuable insights about the series. By considering the various time scales of the series, significant information can be extracted from the signals, which represent the raw data (52).

In order to explore the interconnection between the prevalence of chronic diseases (diabetes, circulatory and respiratory diseases) and population ages 65 and above in terms of both frequency and time, we utilize wavelet coherence analysis with Morlet’s specification, as explained in Macedo (53). This analysis allows us to examine the joint behavior of time series. The Morlet wavelet used in this analysis is defined in Equation 5:

(5) $$\psi^{M}\left(t\right)=\frac{1}{\pi^{1/4}}e^{i\omega_{0}t}e^{-t^{2}/2}$$

Where ω_0_ is the central frequency of the wavelet (we have settled it to 10).

The continuous wavelet transformation is given by Equation 6, following (54, 55):


(6) $$W_{x}\left(u,s\right)=\int s\left(t\right)\frac{1}{\sqrt{s}}\psi \left(\frac{t-u}{s}\right)dt$$

*W_x_ (u,s)* is calculated by projecting the specific wavelet *ψ* (·) onto the selected time series, as stated in (52).

To explore the simultaneous patterns of time and frequency in the relationship among circulatory, respiratory and diabetes appearance and the percentage of older adult population, we used the wavelet-squared coherence. Prior to delving into this analysis, it is necessary to introduce the cross-wavelet transformation. In accordance with the methodology proposed by (56), the cross-wavelet transform for the signals *x (t)* and *y (t)* can be expressed as follows:


(7) $$W_{x,y}\left(u,s\right)=W_{x}\left(u,s\right){W^{*}}_{y}\left(u,s\right)$$

In this context (Equation 7), the position is represented by the symbol ‘*u*’, the scale is denoted by ‘*s*’, and the complex conjugate is indicated by the asterisk symbol ‘*’.

Torrence and Webster (57) provide an explanation of the squared wavelet coefficient, which is defined as the area in the time-scale space where the time series exhibits a significant level of shared power. The squared wavelet coefficient is defined in Equation 8.


(8) $$R^{2}\left(u,s\right)=\frac{{\left|S\left(s^{-1}W_{x,y}\left(u,s\right)\right)\right|}^{2}}{S\left(s^{-1}{\left|W_{x}\left(u,s\right)\right|}^{2}\right)S\left(s^{-1}{\left|W_{y}\left(u,s\right)\right|}^{2}\right)}$$

The smoothing parameter, denoted as “*s*,” determines the value of squared.

The range of wavelet coherence is from 0 to 1, denoted as *R^2^ (u, s)*. When the value is near zero, it indicates a weak interdependence, and vice versa. By evaluating the value of Equation 8, we can analyze and differentiate the level of interdependence between the prevalence of chronic diseases (diabetes, circulatory and respiratory diseases) and Population ages 65 and above by comparing correlations across different time frames. The scatter plots of the continuous wavelet coherence are exposed and explained in the results section.

In Table 1 we have exposed the source and explanation of each data set used in the analysis.

## 4 Results

From the descriptive statistics listed in Table 2, the minimum, maximum and average values of the data explained in Table 1 and the standard deviation of each data set can be obtained. For example, the data on the total number of patients with respiratory diseases show that the maximum is 1,360,673 and the minimum is 2,170; while for patients with circulatory system diseases, the maximum number of patients per year in European countries is 3,100,387, while the minimum is 2,366. The annual maximum number of patients with diabetes is 233,469 and the minimum is 165. When analyzing the health infrastructure, the average number of beds per thousand people is 2 and the maximum is 9. The proportion of the population over 65 years old in the EU is at least 10 and at most 24, with an average of 17.07%.

**Table 2:** Descriptive statistics.

	*N*	Minimum	Maximum	Mean	Std. Deviation
GDP	594	4.3200	4259.9300	499.762357	817.0104890
INF	594	−4.5000	45.7000	2.496128	3.2548009
UR	594	1.8100	27.4700	8.556263	4.3340007
HS/GDP	580	4.2100	12.8200	8.014483	1.8134861
HOSP-B	457	2.0500	9.1200	5.444311	1.7732501
Respiratory diseases	535	2,170	1,360,673	223495.39	284690.587
Circulatory diseases	535	2,366	3,100,387	413863.11	623713.538
Diabetes Mellitus	535	165	233,469	31149.81	47373.113
POP65	594	9.9400	23.6800	17.069478	2.8326732
Valid *N* (listwise)	390				

According to the extended Dickey-Fuller test: For the older adult population, the *p*-value of the percentage of the population over 65 years old does not exceed 0.05 for any differentiation (Table 3), so we cannot reject the null hypothesis and conclude that the time series is not stationary. At the first differentiation, the *p*-value of the percentage of the population over 65 years old is less than 0.05, so we can reject the null hypothesis and conclude that the time series is stationary. For any differentiation, the p-value of the number of patients with respiratory diseases is not less than 0.05 (Table 3), so we can reject the null hypothesis and conclude that the time series is stationary. For any differentiation, the p-value of the number of patients with diabetes is not less than 0.05, so we can reject the null hypothesis and conclude that the time series is stationary. The *p*-value for the number of patients with circulatory diseases is lower than 0.05 (undifferentiated). Therefore, we can reject the null hypothesis and conclude that the time series is stationary (Table 3).

**Table 3:** Augumented Dickey Fuller test: older adult population (POP65).

Variable/method	*t*-Statistic	Prob.*
Older adult population (POP65)	−1.462618	0.5520
Older adult population (POP65) (first difference)	−10.4928	0.0000
Respiratory diseases	−27.56091	0
Diabetes diseases	−11.20367	0.0000
Circulatory diseases	−27.18805	0.0000

The *p*-value for the effect of the number of patients with circulatory diseases on the residuals of the equation is higher than 0.05, so we conclude that there is no evidence of heteroskedasticity (Table 4). Since the *p*-value for the effect of the number of patients with diabetes on the residuals of the equation is lower than 0.05, we conclude that there is no evidence of heteroskedasticity. To reduce heteroskedasticity between the data, we have applied logarithm on the diabetes data, thereby eliminating heteroskedasticity. In addition, the *p*-value for the logarithm of the number of patients with diabetes on the residuals of the equation is higher than 0.05, so we conclude that there is no evidence of heteroskedasticity (Table 4). For the effect of the number of patients with respiratory diseases on the residuals of the equation, the *p*-value is higher than 0.05, so we conclude that there is no evidence of heteroskedasticity.

**Table 4:** Heteroskedasticity tests.

	Heteroskedasticity test	*F*-statistic	Obs*R-squared	Prob. *F* (1,533)	Prob. Chi-Square (1)
Circulatory diseases – older adult population percentage	Glejser	0.323583	0.324601	0.5697	0.5689
Diabetes Mellitus – older adult population percentage	Glejser	11.73316	11.52352	0.0007	0.0007
Log Diabetes Mellitus – older adult population percentage	White	5.375579	10.59762	0.0049	0.005
Respiratory diseases – older adult population percentage	Glejser	2.409903	2.408058	0.1212	0.1207

Table 5 presents the Pearson correlations showing significant parametric correlations between respiratory and circulatory diseases in each country, with a correlation of 0.955 at the 1% significance level.

**Table 5:** Pearson correlations.

	GDP	INF	UR	HS/ GDP	HOSP-B	Respiratory diseases	Circulatory diseases	Diabetes Mellitus	POP65
GDP	1	−0.148^**^	−0.018	0.562^**^	−0.039	−0.013	−0.008	0.807^**^	0.354***
INF		1	−0.079	−0.339^**^	0.120^*^	0.060	−0.035	0.045	−0.222**
UR			1	−0.108^**^	−0.025	0.086^*^	0.083	−0.049	−0.026
HS/GDP				1	−0.084	−0.136^**^	−0.135^**^	0.315^**^	. 442***
HOSP-B					1	0.001	0.001	0.001	0.147***
Respiratory diseases						1	0.955^**^	0.060	−0.248***
Circulatory diseases							1	0.017	−0.295***
Diabetes Mellitus								1	−0.210***
POP65									1

Source: Authors’ processing, ***, ** and * denote significance at 1, 5 and 10 percent level, respectively.

The proportion of population aged 65 years and above is significantly negatively correlated with diabetes, number of respiratory and circulatory diseases, and inflation, and significantly positively correlated with GDP, number of hospital beds (HOSP-B), and health expenditure as a percentage of GDP (HS/GDP) (significance level 1%). Annual diabetes cases are also significantly positively correlated with GDP *per capita*.

In order to outcome the best results we have logged the GDP *per capita*, expressed in Purchasing Power Parity, in order to see the effect of the change in economic growth, expressed by the growth in GDP, on the change in the percentage of population over 65. Therefore, the effect of the logged GDP is significantly positive over the change in percentage of older adult population, as shown in Table 6 (with a unit change of 2.858 in older adult population percentage at every one-unit change in logged GDP, significance at 1%). The unemployment rate presents a negative significant *β*, as it has a negative effect of 0.072 units of change of older adult population percentage of total population, with a significance level of 1%. Negative effect on change in older adult population percentage has also the annual inflation, with an effect of 0.262, being highly significant, with a significance level under 1%. In awareness of the health infrastructure, both the logarithmic number of hospital beds reported to 1,000 people and the spending percentage in health as reported to GDP have a positive, significant impact on percentage of older adult population over 65, with effects of 0.244 and 0.578, at 0.01 significance levels (Table 6).

**Table 6:** Results from the GMM model.

Dependent variable: population ages 65 and above-percent of total (POP65)	*β*
GDP	2.858***
UR	−0.072***
INF	−0.262***
HOSP-B	0.244***
HS/GDP	0.578***
Diabetes Mellitus	−1.533***
Circulatory diseases	−4.126***
Respiratory diseases	−2.446***
Constant	34.04***
R-squared	0.4034
Observations	565
No. of countries	27
AR(1) test (*p*-value)	0.045
AR(2) test (*p*-value)	0.012
Hansen test (*p*-value)	0.243
Chi^2^ test (*p*-value)	0.000

Source: Authors’ processing. *** denotes significance at 1 the percent level.

When analyzing the impact of circulatory, respiratory and diabetes health occurrences over the change in older adult population percentage, results from the general method of moments show significant negative β coefficients (respectively one unit of change in diabetes, respiratory and circulatory number of occurrences have an effect of −1.533, −4.126 and −2.446 units of change in older adult people percentage of total population).

Then Hansen test results are under the threshold of 0.25, according to Roodman statement (58). The R squared has a value of 0.4. The Arellano-Bond test for autocorrelation AR (1) and AR (2) results show that the idiosyncratic error term is serially correlated, as the values are under 0.5. We are rejecting the null hypothesis of no first order serial correlation and second order serial correlation. The chi square results show that there is a significant relationship between the variables involved in the model, as the associated *p*-value is under 0.05.

Since our data is not stationary, we estimate and plot the wavelet coherence for each stock analyzed. As stated in Labat (59): “Continuous wavelet cross-correlation provides the temporal distribution of the correlation between two signals, while continuous wavelet coherence provides a qualitative estimate of the temporal evolution of the degree of linearity of the relationship between the two signals on a certain scale.”

**Figure 1:**
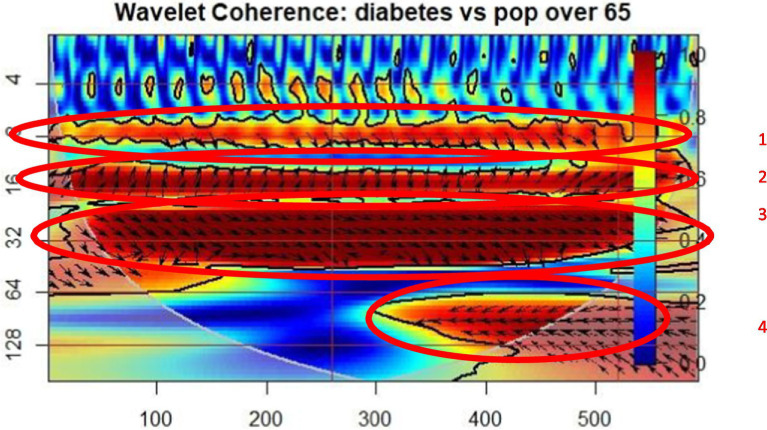
Wavelet coherence: Diabetes Mellitus – population over 65 (POP65).

In Figure 1, the wavelet coherence for the diabetes patients’ occurrences and the percentage of the older adult persons from total is plotted. It can be observed, in the cone of influence, that there are four regions of coherent oscillation behavior, firstly between 7 and 9 period coherence lags, in the whole period of time analyzed. In the first significant coherence lag, mentioned before, the arrows direction points to a lagging A index, that means that in the older adult people percentage (B) determined the diabetes occurrences (A), with a lag of 7 to 9 periods, where period means 2 months. Also, in the second coherence region, the variables are in phase, the diabetes occurrences are leading the older adult people percentage, with a lag of 16 periods. In the third significant coherence region, the coherence region shows that the older adult people percentage is leading the diabetes occurrences, with a lag of 20 to 64 periods. Also, in the fourth region, the diabetes occurrences are lagging, meaning that the older adult people percentage influence the diabetes occurrences, with a lag of 64 to 128 periods.

**Figure 2:**
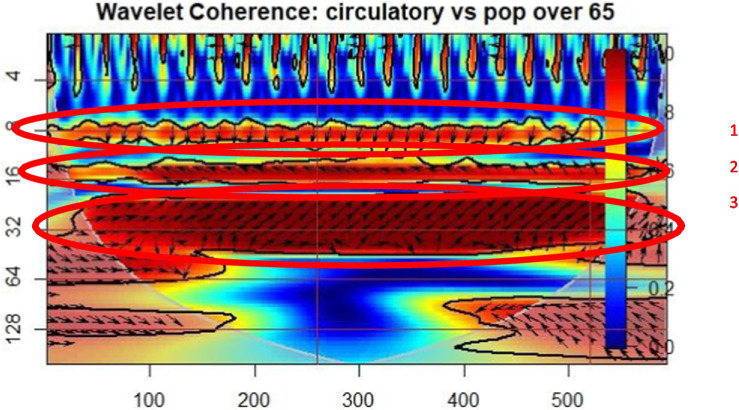
Wavelet coherence: circulatory diseases – population over 65 (POP65).

In Figure 2, the wavelet coherence between the occurrence of patients with circulatory diseases and the percentage of people over 65 is exposed. Three regions of coherence oscillation behavior can be observed in the cone of influence. In the first significant coherence regions, the arrows direction points to a lagging A index, that means that in the older adult people percentage (B) determined the circulatory occurrences (A), with a lag of 7 to 9 periods, where period means 2 months. In the second coherence period, the occurrence of circulatory diseases is lagging, thus the older adult people percentage is determining the circulatory patient’s occurrence, with a lag of 16 periods. In the third coherence region, the circulatory disease occurrence is leading the percentage of the older adult population, with a lag of 32 periods.

**Figure 3:**
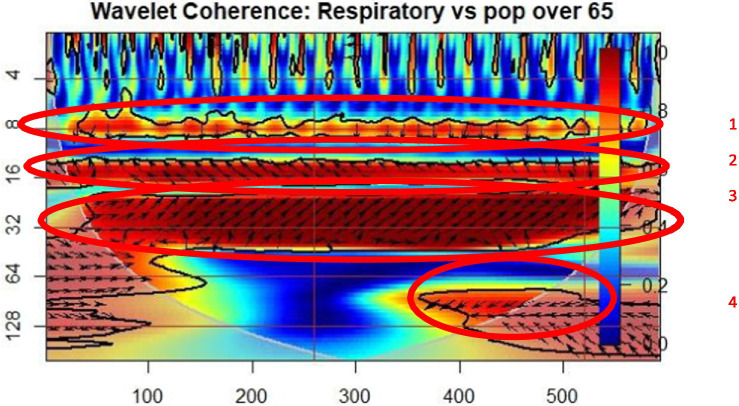
Wavelet coherence: respiratory diseases – population over 65 (POP65).

Figure 3 shows the wavelet relationship between the incidence of patients with respiratory system diseases and the proportion of people over 65 years of age. Four regions where coherent oscillatory behavior can be observed in the influence cone. In the first area of significant coherence, the direction of the arrow points to the lagged A index, i.e., in older adults the percentage (B) determines the occurrences of the patients with respiratory diseases (A) delayed by 7 to 9 cycles, where period means 2 months. In the second coherence period, the onset of respiratory disease is delayed, so the proportion of older adults delayed by 16 cycles determines the onset of circulatory disease. In the third continuity area, the incidence of respiratory diseases is among the highest in the older adult population proportion, with a delay of 32 cycles. Also in the fourth region, the onset of respiratory was delayed, meaning that the percentage of older adults affected the onset of respiratory patients by 64 to 120 periods.

## 5 Discussion

Several important findings arise from our analysis. We mention that the study’s findings align with the existing literature, even though those articles individually addressed the impact of various variables on the older adult population.

### 5.1 Economic factors and older adult population

The results based on system GMM model reveal that increasing GDP has a positive and significant impact on Population ages 65 and above in the sample countries. These results are in line with several studies, such as those conducted by Gildas et al. (14), Galappaththi et al. (15), McMaughan et al. (10) and Cravino et al. (16), which found similar results between healthcare infrastructure and economic stability in older adult populations. Additionally, Tang et al. (28) highlighted the importance of adequate health financing and accessible healthcare services in managing chronic diseases among the older adult. An increase in Gross Domestic Product (GDP) indicates a country’s economic expansion, signifying improved national economic health and wealth. This growth has multifaceted impacts, notably on the demographic composition, particularly elevating the segment aged 65 and above (*Improved Healthcare Access and Quality*) (60). A flourishing economy boosts healthcare access and quality by enabling more substantial investments in healthcare infrastructure, services, and innovations. This enhancement in medical care, including preventive and chronic disease treatments, directly extends life expectancies, swelling the older adult population’s proportion. Economic prosperity also elevates living standards across the board—better nutrition, housing, and safer working conditions contribute to healthier, longer lives (*Enhanced Living Standards*) (16). Additionally, with more resources at its disposal, a government can allocate more to pensions, social services, and eldercare, ensuring the older adult enjoy a higher quality of life and financial security (14). Economic growth contributes to *reducing poverty and inequality*, directly correlating with fewer health issues and longer lives. Moreover, a higher GDP encourages *advancements in medical technology and research*, leading to breakthrough treatments and disease management strategies that benefit the older adult. Collectively, these factors intertwine to foster a demographic shift toward an older population as the nation’s economy grows.

According to the presented system GMM model, the variables unemployment rate and inflation have a significant negative impact on the population aged 65 and above (POP65) in the analyzed countries. These results are in line with several studies (44–47). Both variables suggest that a stable economy, characterized by low unemployment and inflation rates, positively contributes to the increase in the proportion of the older adult population by improving living conditions and access to resources for this age group.

A reduction in unemployment and inflation reflects a stable, thriving economy, greatly benefiting those aged 65 and above. Such stability boosts employment, thereby enhancing pension and social security contributions, which fortifies the financial safety net for the older adult. Improved pensions and benefits from this reinforced support system elevate the quality of life and lifespan for seniors (4). Economic growth, accompanied by low unemployment and inflation, also spurs healthcare investments, leading to better medical service access and quality for the older adult. This includes comprehensive care from preventive measures to chronic disease management and advanced treatments, significantly lowering mortality rates among seniors and increasing their demographic share. Moreover, low inflation helps stabilize living costs, crucial for the older adult relying on fixed incomes, ensuring they can afford healthcare and essentials for a healthy life (5). This economic stability, encouraging savings and investments, supports the older adult’s financial security, thereby contributing to their well-being and a rise in the older adult population, illustrating the positive effects of economic stability and growth on senior citizens’ longevity and quality of life (19).

Therefore, governments must integrate economic policies promoting growth and stability with targeted investments in healthcare, social services, and pensions to improve the older adult’s quality of life. This approach will help address the demographic shift, ensuring economic prosperity enhances healthcare access, financial security, and overall well-being for senior citizens, while reducing the impact of economic fluctuations on vulnerable groups.

### 5.2 Healthcare capacity and older adult population

In the GMM models, we find that an increase in healthcare capacity, as measured by hospital beds and healthcare spending as a percentage of GDP, has a positive and significant effect on the proportion of the population aged 65 and above. This result suggests that well-developed healthcare systems contribute to a higher life expectancy and better health outcomes for the older adult, enabling them to live longer and healthier lives.

This result is consistent with previous studies that have shown that improved healthcare infrastructure and increased health spending are associated with lower mortality rates and improved quality of life among older adults (1, 61). Moreover, studies such as those by Holecki et al. (10) highlight the importance of healthcare capacity in managing the needs of an aging population, especially in countries with a high proportion of older adult individuals.

Boosting hospital bed numbers significantly impacts the older adult population by improving access to healthcare and cutting down on treatment delays, crucial for those prone to chronic conditions. This availability allows for prompt medical interventions, enhancing recovery chances and health outcomes for the older adult (33). Enhanced care capacity meets seniors’ health needs and boosts their well-being by reducing waiting periods for vital surgeries and treatments, key to preserving their health. Moreover, more hospital beds typically indicate wider healthcare infrastructure and service improvements, elevating care quality. Such advancements lower mortality rates in the older adult, supporting a larger, healthier aged population.

Rising health spending reflects a commitment to enhancing healthcare services and innovation, crucially benefiting the older adult. This investment means greater access to quality care, from preventive measures to cutting-edge treatments, essential for addressing the complex health needs of seniors. Improved healthcare facilitates better chronic disease management, fewer complications, and more accessible treatments for urgent health issues (35). Consequently, such advancements lead to longer life spans for the older adult by promoting earlier disease detection and more effective care, thereby supporting a significant increase in the population aged 65 and above through improved health and well-being.

### 5.3 Chronic disease (such as the prevalence of Diabetes Mellitus, diseases of the circulatory and respiratory systems) and older adult population

The results of our study reveal negative associations between Chronic Disease and the Older Adult Population, which coincide with previous studies (24, 25, 42) indicating that the prevalence of conditions such as diabetes, cardiovascular, and respiratory diseases contributes to increased mortality and reduced life expectancy among older adults. These findings align with prior research demonstrating that chronic diseases significantly impact longevity and quality of life in aging populations (44–47), emphasizing the need for effective management and prevention strategies.

A surge in hospitalizations for Diabetes Mellitus, circulatory, and respiratory conditions often mirror an expanding older adult population. These ailments are predominantly seen in older individuals, with aging contributing to the body’s decreased disease resistance. As the older adult segment enlarges, the frequency of these chronic diseases escalates, leading to an increased demand for hospital-based care and management. This pattern of hospital admissions serves as an indicator of the rising numbers within the older adult demographic, highlighting the intensified requirement for healthcare services to cater to the specific, age-related health challenges they face (39–41).

The wavelet coherence graphs indicate a significant relationship between the prevalence of chronic diseases, such as diabetes, circulatory, and respiratory conditions, and the proportion of the older adult population (POP65). These chronic diseases, due to their severity and associated complications, directly influence mortality and life expectancy among older individuals, creating a vicious cycle of unhealthy aging.

### 5.4 Policy recommendations

Policy Recommendations for Managing the Impact of Chronic Diseases on the Older Adult Population are directly related to healthcare capacity and economic indicators because an adequate healthcare infrastructure is essential for providing comprehensive care and management of chronic conditions prevalent among the older adult. By implementing targeted recommendations (such as: improving healthcare infrastructure, promoting economic stability and adequate health financing, and enhancing the accessibility and expansion of healthcare services), policymakers can better address the economic and healthcare challenges posed by chronic diseases, reducing their impact on healthcare expenditures and enhancing the resilience and sustainability of healthcare systems and societies. Policy recommendations should focus on enhancing healthcare infrastructure by investing in facilities and equipment to support the growing needs of the older adult and expanding preventive care programs to reduce the incidence and severity of chronic diseases. Additionally, it is essential to strengthen primary care systems, support the healthcare workforce, promote economic stability for consistent healthcare funding, and increase access to social support services to ensure comprehensive care for aging populations.

## 6 Conclusion

This research embarked on an ambitious journey to unravel the complex interplay between healthcare system capabilities, economic indicators, prevalence of Chronic Disease and their impact on the proportion of the older adult population in European Union member countries. Through a meticulous examination utilizing System GMM and wavelet coherence analysis over a period of 21 years, this study has illuminated the dynamic relationships that underpin the demographic structure of the EU, particularly focusing on those aged 65 and above.

The study highlights that all three main categories of factors influence the proportion of the older adult population (POP65) in European countries: economic factors, healthcare system capacity, and health-related variables. Economic factors, such as GDP, unemployment, and inflation, play a crucial role in determining the resources available for older adult care. The capacity of the healthcare system, measured by the number of hospital beds and healthcare expenditure as a percentage of GDP, is essential for providing adequate care and managing chronic diseases. Finally, health-related variables, including the prevalence of chronic conditions such as diabetes, circulatory diseases, and respiratory diseases, have a significant negative impact on the proportion of the older adult population, as they increase mortality and reduce life expectancy.

Moreover, the study’s innovative use of wavelet coherence analysis provided further insights into the temporal and frequency-dependent correlations between the health-related variables and the older adult population proportion. This analysis highlighted how fluctuations in healthcare and economic variables synchronously affect the demographic structure over time, offering a nuanced understanding of the dynamic forces at play.

The research findings advocate for the necessity of integrated economic and healthcare policies aimed at enhancing the welfare of the older adult in the EU. The study’s insights into the critical importance of economic and healthcare system robustness in supporting the aging population are imperative for policymakers and healthcare planners. Addressing the challenges posed by an aging demographic requires a holistic approach, ensuring sustainable development and the well-being of the older adult in society.

One limitation of the study is its reliance on aggregate data from European Union countries, which may obscure specific national differences in healthcare systems and economic conditions that could differently influence the older adult population’s proportion.

Future research. To refine our understanding of the mechanisms at play, future research should investigate the differential impacts of healthcare quality and economic changes on subpopulations within the older adult, considering variables such as socio-economic status, gender, and regional disparities across the European Union.

## Data Availability

The original contributions presented in the study are included in the article/supplementary material, further inquiries can be directed to the corresponding author.
